# Sinomenine ameliorates lipopolysaccharide-induced acute lung injury by stimulating M2 polarization and suppressing pyroptosis in alveolar macrophages

**DOI:** 10.1016/j.clinsp.2025.100832

**Published:** 2025-11-19

**Authors:** Xiaoli Jian, Liang Zhao, Jiawu Yang, Peilong Li, Chuxiong Gong, Qiong Li, Zhu Tian, Enli Lan, Tingyun Yuan, Feng Li

**Affiliations:** Department of Pulmonary and Critical Care Medicine, Kunming Children’s Hospital, Kunming, China

**Keywords:** Sinomenine (SINO), Acute lung injury (ALI), Alveolar macrophages (AMs), Protective effect

## Abstract

**Objectives:**

Alveolar Macrophages (AMs), key defenders against Acute Lung Injury (ALI), are a potential therapeutic target. Sinomenine (SINO) has shown protective effects against ALI. This study investigates whether SINO exerts its protection by modulating AM polarization and pyroptosis.

**Method:**

39 mice were divided into three groups: control, Lipopolysaccharide (LPS), and ALI+SINO groups. After administration of vehicle and SINO (60 mg/kg) for 1 h, mice were intratracheally injected with LPS at 10 mg/kg to induce ALI. A separate cohort received 20 mg/kg LPS for survival analysis. The survival rate, pathological alteration, lung Wet/Dry weight (W/D) ratio, and Myeloperoxidase (MPO) activity in the lung, as well as neutrophil count and protein content in Bronchoalveolar Lavage Fluid (BALF), were assessed in mice. Cytokine levels in BALF, serum, and AMs were measured using Enzyme-Linked Immunosorbent Assay (ELISA). The polarization type of AMs was determined by flow cytometry, western blot, and immunofluorescence analysis. Pyroptosis-associated molecules were determined by western blot.

**Results:**

SINO significantly improved the survival rate and lung pathological alterations in ALI mice and reduced the W/D ratio, protein concentration, MPO activity, and inflammation of lung tissue. Both *in vivo* and *in vitro* analysis demonstrated that SINO could suppress pyroptosis and induce M2 polarization in LPS-induced AMs. Mechanistically, SINO repressed the LPS-activated NF-κB pathway.

**Conclusions:**

The present findings illustrate that SINO exerts a protective effect on LPS-induced ALI via stimulating M2 polarization and suppressing pyroptosis in AMs, and its mechanism is related to the regulation of the NF-κB signaling pathway.

## Highlights

• LPS stimulation led to systemic inflammation in mice.
• SINO could suppress pyroptosis and induce M2 polarization in LPS-induced AMs.
• SINO repressed the LPS-activated NF-κB pathway.

## Introduction

Acute Lung Injury (ALI) is an acute bilateral pulmonary infiltration disease characterized by a strong inflammatory response in the air spaces and lung parenchyma, which finally leads to the impairment and even loss of lung function^1^. Multiple factors, including severe sepsis, pneumonia, severe trauma with shock, as well as aspiration of harmful gas, could cause ALI. Despite the pathogenesis and treatment of ALI having been intensively studied for decades, its mortality remains alarmingly high (approximately 40 %)^2^. Unfortunately, to date, no specific pharmacotherapy has been shown to effectively treat ALI. Hence, the exploration of novel treatment strategies for patients with ALI is an urgent need.

Since dysregulated inflammation is a core issue in ALI^3^, limiting excessive inflammation has been considered a promising strategy for treating ALI. During ALI, Alveolar Macrophages (AMs) are well known to initiate inflammatory responses in the early phase and promote tissue repair in the resolution phase^4^. Increasing evidence supports that the phenotype of AMs, which includes classically activated phenotype (M1) and alternatively activated states (M2), is closely related to inflammation during ALI^5^^,^^6^. The shift between M1 and M2 polarization of macrophages is dependent on microenvironmental stimuli and has been implicated in the progression of ALI. At the initial acute phase of ALI, M1-polarized AMs release pro-inflammatory cytokines to stimulate the alveolar cells and recruit neutrophils to the alveolar space, thereby enhancing the inflammatory response for the elimination of pathogens^5^. At the resolution phase, M2-polarized AMs are activated, secreting anti-inflammatory cytokines to promote tissue remodeling^5^. The balance between M1/M2 phenotypes of AMs is deeply correlated with the severity of lung injury, which might be a promising target for the treatment of ALI^7^.

Multiple previous studies presumed that the uncontrolled inflammation in ALI may result from the release of proinflammatory cytokines during immune cell death^8^. Pyroptosis is an inflammatory programmed cell death mediated by the inflammasome, which could cause a robust inflammatory response to defend against infections^9^. Prior to pyroptosis, NLRP3 is upregulated and then forms a protein complex with ASC and pro-Caspase-1, thus triggering pyroptosis. A rupture of the cytoplasmic membrane resulting from pyroptosis leads to the release of proinflammatory cytokines, including Interleukin (IL)-18 and IL-1β, followed by excessive tissue inflammation. Recently, several studies revealed that various inflammatory diseases, including ALI^10^. involve the dysregulated activation of NLRP3 inflammasome. The pyroptosis of AMs has been reported to disrupt the lung homeostasis by causing excessive production of proinflammatory cytokines, thus contributing to the progression of ALI^11^. In consequence, suppressing AMs pyroptosis may be another potential target for therapeutic intervention of ALI.

**Fig. 1:**
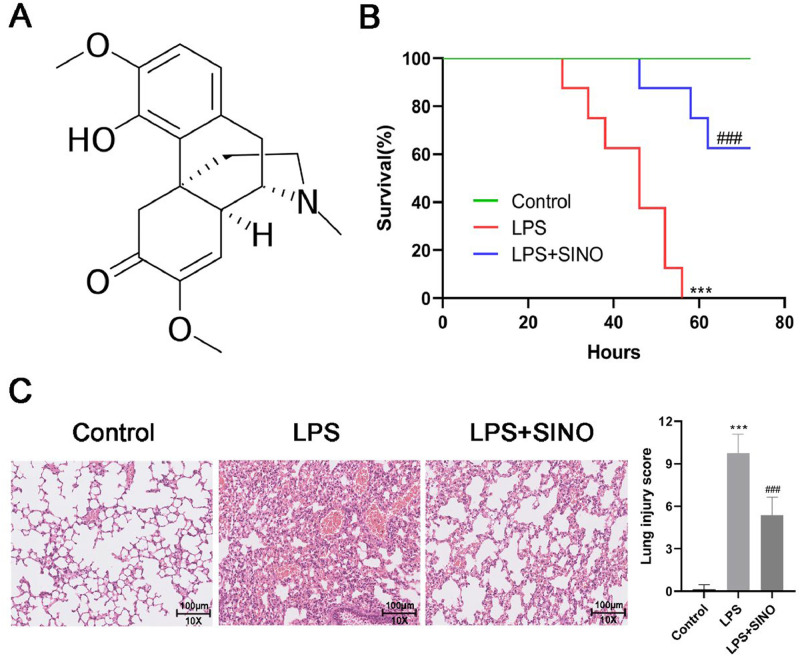
SINO ameliorated survival and lung histopathologic change of LPS-induced ALI mice. (A) Chemical structure of SINO. (B) Survival curve for mice from diverse groups. Both the LPS and LPS+SINO groups received LPS (20 mg/kg; a lethal dose), and the LPS+SINO group given SINO (100 mg/kg) 1 h before LPS challenge. Mice without LPS stimulation were regarded as the control. (*n* = 8 per group). Mice were intratracheally administered with LPS (10 mg/kg) or PBS, half of the mice exposed to LPS were pre-treated with SINO (100 mg/kg i.p.); a series of analyses were performed after 24 h LPS stimulation (*n* = 5 per group). (C) Representative images of H&E staining for lung tissue (Scale bar: 100 μm). (D) Quantitative assessment of lung injury score using a five-item histological scoring system. Each feature (alveolar disruption, inflammatory infiltration, edema, hemorrhage, and exudate) was graded 0‒4. The final score was calculated as the sum of the five scores per field, averaged across five fields per mouse. *** *p* < 0.005 vs. control group, ^###^*p* < 0.005 vs. LPS group.

As a widely studied natural alkaloid with potent anti-inflammatory activity, Sinomenine (SINO; 9α,13α,14α−7,8-Didehydro-4‑hydroxy-3,7-dimethoxy-17-methylmorphinan-6-one) (Fig. 1A) is derived from the plant *sinomenium acutum*, which is widely applied in rheumatoid arthritis therapy^12^. During the last decade, research on the potential of SINO for improving ALI has gradually aroused great interest. Liu et al. proved that the SINO exerted a protective effect on the *Escherichia coli*-induced ALI mouse model by alleviating inflammatory cytokine production and oxidative stress in the lung^13^. A recent study also revealed that SINO can significantly attenuate the degree of injury, inflammatory cytokine secretion, and oxidative stress in septic-induced ALI^14^. Nevertheless, the mechanism behind this effect is largely unclear.

Herein, the authors explored the effects and underlying mechanism of SINO on LPS-induced ALI. The authors show for the first time that the effect of SINO protecting against ALI is associated with its regulatory role in the polarization and pyroptosis of AMs. Moreover, the authors also revealed that the downregulation of the NF-κB signaling pathway would be a possible mechanism underlying the action of SINO in ALI.

## Materials and methods

### Lipopolysaccharide (LPS)-induced ALI mouse model and treatment

A total of 39 C57BL/6 mice (male; 18‒22 g) purchased from Guangdong Medical Laboratory Center (Guangzhou, China) were randomly divided into three groups (*n* = 13) as follows: 1) Control; 2) LPS; 3) LPS+SINO groups. ALI was induced by the intratracheal administration of LPS (Sigma, MO, USA; cat.no#L4391) (10 mg/kg, dissolved in PBS)^15^. Meanwhile, mice from the control group were intratracheally given equal volumes of the vehicle (PBS).

Previous *in vivo* studies demonstrated that SINO at a concentration of 100 mg/kg effectively protected against LPS-induced ALI^13^^,^^16^. SINO (100 mg/kg, dissolved in saline plus DMSO solution) was intraperitoneally administered 1 h prior to the LPS inhalation. Mice from the control and LPS groups simultaneously received intraperitoneal injection of saline plus DMSO solution. 12 h after LPS inhalation, the lung tissues, Bronchoalveolar Lavage Fluid (BALF), and serum were harvested for further analysis. 10 mg/kg of LPS was used to induce ALI in the main experiment. For survival analysis, an independent cohort of mice was intratracheally administered a higher dose of LPS (20 mg/kg) to induce lethal ALI. These mice were monitored every 8 h for up to 72 h post-LPS challenge^15^. At the experimental endpoint, all mice were deeply anesthetized by intraperitoneal injection of pentobarbital sodium (150 mg/kg) and euthanized via exsanguination. All experimental procedures regarding animals were approved by the Institutional Animal Care and Use Committee of Kunming Children’s Hospital (Approval number: Kmmu20241479) and followed the ARRIVE guidelines.

### Histological assessments

To investigate the histological alterations, the harvested lung tissues were subjected to fixation, sectioning, and staining with Hematoxylin/Eosin (H&E) in accordance with a standard protocol. Finally, the stained sections were observed by the light microscope (Olympus). Lung injury was semi-quantitatively scored based on five key pathological features, as previously described:^17^ 1) Alveolar structure damage, 2) Inflammatory cell infiltration, 3) Interstitial edema, 4) Hemorrhage, and 5) Hyaline membrane formation. Each parameter was graded on a scale from 0 (absent/normal) to 4 (severe). For each animal, five randomly selected high-power fields (HPFs, 400 ×) were scored, and the average of all scores was calculated to generate a final cumulative injury score (maximum possible score = 20). Scoring was performed independently by two blinded investigators.

### Measurement of lung water content

Lung Wet/Dry weight (W/D) ratio was calculated to investigate the water content in the lung, thereby evaluating the degree of pulmonary edema of mice from each group. Briefly, the harvested lung was weighed and dried at 55 °C for 48 h before being weighed once more when it was dry.

### Myeloperoxidase (MPO) measurement

MPO activity in the lung, an index of neutrophil infiltration^18^, was spectrophotometrically assessed using the Mouse MPO ELISA Kit (LifeSpan BioSciences, WA, USA; cat.no#LS-F24875) in this study. Briefly, the collected lung tissues were homogenized on ice using a homogenizer. The homogenate was subjected to centrifugation to obtain the supernatant. The MPO activity of the lung tissues was estimated by the test kit as per the manufacturer's protocol.

### BALF collection and analysis

After endotracheal intubation, ligation of the left main bronchus was carried out, followed by lavaging of the right lung three times using PBS (0.5 mL × 3) to collect BALF. After the centrifugation of BALF samples, the precipitate was resuspended in saline for neutrophil counting, while the cell-free supernatants were harvested to detect the total protein contents and cytokine concentrations in BALF. The collected cells were subjected to Wright-Giemsa staining, followed by neutrophil counting under a light microscope. The total protein in BALF was determined by using the Bicinchoninic Acid (BCA) Protein Assay Kit (Beyotime, China).

### Blood collection

Blood was collected from the abdominal aorta of mice before sacrifice, and subsequently centrifuged to collect serum. The serum was maintained at −80 °C for the subsequent determination of cytokines.

### AMs isolation

The isolation of AMs from BALF was conducted as previously described^15^. Briefly, the harvested BALF was subjected to centrifugation to obtain pellet. Next, the BALF pellet was resuspended and then incubated with RPMI 1640 medium containing 10 % Fetal Bovine Serum (FBS), 100 IU/mL penicillin and 100 µg/mL streptomycin for 1 h. Finally, the supernatant was discarded, and adherent cells were identified as AMs.

### Flow cytometer (FCM)

To determine the polarization of AMs from diverse groups, FCM using F4/80^+^/iNOS^+^ or F4/80^+^/CD206^+^ staining was performed. Initially, to enhance the specificity of the antibody, the isolated AMs from the BALF were incubated with FcR Blocking Reagent (Miltenyi, Auburn, *CA*, USA). After staining with surface markers (F4/80), AMs were fixed and permeabilized, followed by staining with the anti-CD206 or anti-iNOS antibody for 30 min. Finally, the percentages of F4/80^+^/iNOS^+^ and F4/80^+^/CD206^+^ were analyzed on flow cytometry (BD Biosciences, *CA*, USA) to determine AMs phenotypes (M1 and M2).

### AMs stimulation and treatment

MH-S, a mouse alveolar macrophage cell line, was obtained from the American Type Culture Collection (VA, USA) to perform *in vitro* study. MH-S cells were seeded in 6-well plates and allowed to adhere to the plates. Before being stimulated with LPS (100 ng/mL) 1 h, cells in the LPS+SINO and LPS groups were pre-treated with an equal volume of SINO (10 μg/mL; dissolved in saline plus DMSO solution) and vehicle, respectively. Cells without LPS stimulation are regarded as the control group.

### Immunofluorescence staining

Following fixation for 1 h, AMs were incubated with primary antibodies against rabbit anti-iNOS at 4 °C overnight for labeling the M1 phenotype of AMs. After being rinsed twice with PBS, AMs were incubated with a FITC-conjugated anti-rabbit IgG for 2 h, followed by incubation with DAPI for 10 min for cell nuclei staining. The stained AMs were imaged using a fluorescence microscope (Leica, Weztlar, Germany).

### Determination of inflammatory cytokines

By using commercially available ELISA kits, levels of pro-inflammatory cytokines (IL-6, IL-1β, TNF-α, and IL-18) in serum, BALF, and supernatants of cultured AMs were detected.

### Western blot

After quantification of the concentration of total protein extracted from lung tissues or AMs, an equal amount of protein was processed for separation by 10 % SDS-PAGE and then transferred to PVDF membranes. Next, the membranes were blocked with skimmed milk for 2 h and subsequently incubated with primary antibodies overnight, followed by incubation with an HRP-conjugated secondary antibody for 1 h. Finally, the bands were developed using an enhanced chemiluminescence detection kit (Millipore, MA, USA). The information of the antibodies used in this study is listed in Table S1.

### Statistical analysis

All the experiments were performed at least three times. The data were expressed as the mean ± standard deviation. One-way ANOVA followed by Tukey’s post hoc test was performed for comparison among multiple groups; *p* < 0.05 was considered significant. All statistical analyses were performed using the GraphPad Prism 7 (GraphPad Software, *CA*, USA).

## Results

### SINO ameliorated LPS-induced ALI in mice

All mice exposed to LPS died within 60 h. The administration of SINO resulted in 62.5 % survival in LPS-induced mice (Fig. 1B). In histopathological analysis, control mice exhibited intact alveolar structures with minimal inflammatory infiltration, whereas LPS-challenged mice showed severe histological damage, including alveolar septal thickening, hemorrhage, and massive leukocyte infiltration. Pre-treatment with SINO markedly alleviated these pathological changes (Fig. 1C). Next, the lung injury was further quantified by a modified Smith-based lung injury scoring system, which showed that LPS induced a significant increase in the lung injury score compared to the control group (Fig. 1D). At the same time, SINO treatment significantly decreased the score (Fig. 1D).

**Fig. 2:**
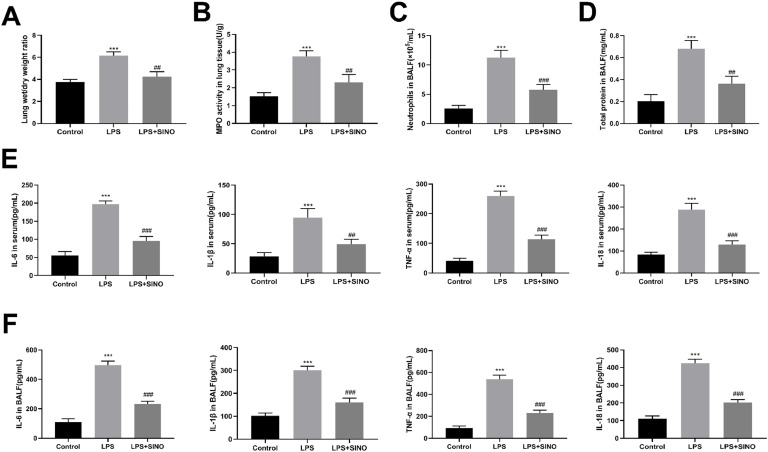
SINO attenuated the inflammation of lung in LPS-induced ALI mice. Mice were intratracheally administered with LPS (10 mg/kg) or PBS, half of mice exposing to LPS were pre-treated with SINO (100 mg/kg i.p.) (*n* = 5 per group). Lungs harvested at 24 h after LPS stimulation were used to determine (A) The lung W/D ratio and (B) MPO activity. (C) The number of neutrophils and (D) Total protein level in BALF. The expression level of IL-6, IL-1β, TNF-α, and IL-18 in (E) Serum and (F) BALF. *** *p* < 0.005 vs. control group, ^###^*p* < 0.005 and ^##^*p* < 0.01 vs. LPS group.

Then, several parameters, such as lung W/D ratio, neutrophil infiltration and total protein content in BALF, and MPO activity in lung tissue, were also detected to further assess the protective effects of SINO on LPS-induced ALI (Fig. 2A‒D). Administration with SINO significantly attenuated LPS-induced pulmonary edema and microvascular permeability, as confirmed by a significant decrease of lung W/D ratio (Fig. 2A), MPO activity in lung tissues (Fig. 2B), number of neutrophils and total protein concentration in BALF (Fig. 2C and D). During ALI progression, pro-inflammatory cytokines, such as IL-6 and IL-1β, have been implicated in the infiltration of neutrophils, thus the authors also investigated the effect of SINO on the release of IL-6, IL-1β, TNF-α, and IL-18 *in vivo* using ELISA kits. Results showed that SINO could successfully suppress the LPS-mediated increased secretion of these cytokines in both serum and BALF from mice undergoing ALI (Fig. 2E and F).

### SINO regulated M1/M2 polarization and suppressed pyroptosis in AMs in LPS-induced ALI

**Fig. 3:**
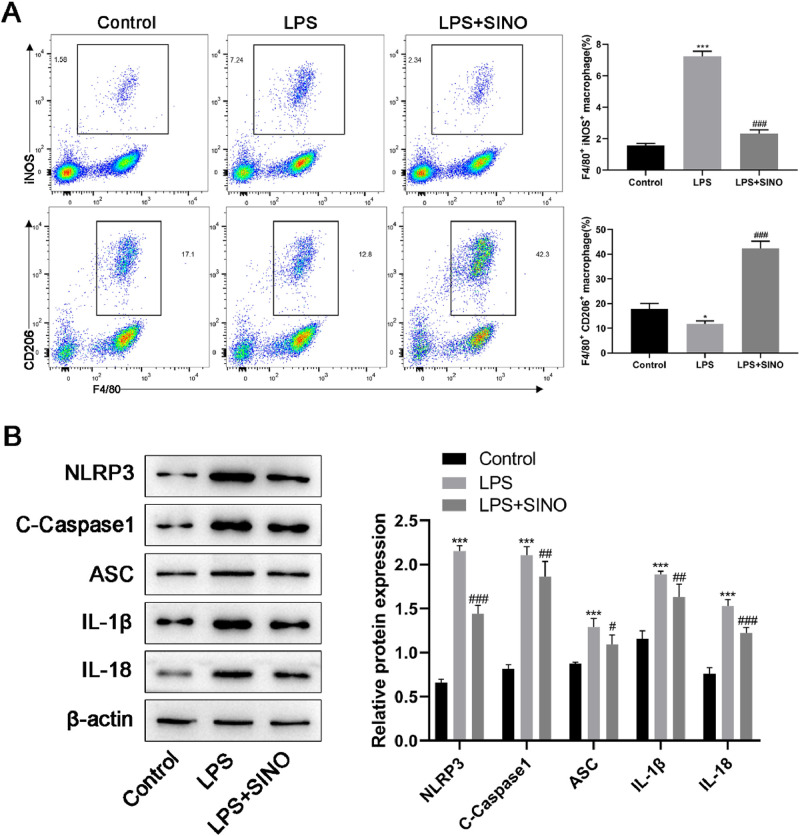
The effects of SINO on the M2 polarization and pyroptosis of AMs *in vivo*. AMs were derived from the BLAF of diverse groups. (A) After AMs were stained with F4/80^+^/iNOS^+^ (M1) and F4/80^+^/CD206^+^ (M2), the expression levels of AMs subsets were analyzed by FCM. The figure right showed the quantitation of the M1/M2 phenotype of AMs from LPS-induced ALI mice. (*n* = 5 per group). (B) The expression levels of pyroptosis related proteins in AMs were detected by western blot. *** *p* < 0.005 and * *p* < 0.05 vs. control group, ^###^*p* < 0.005 and ^##^*p* < 0.01 vs. LPS group.

M1/M2 polarization of macrophages is well-known to be closely related to the inflammatory response. Recently, AMs are reported to polarize toward the M1 phenotype during the course of LPS induced ALI^19^. Therefore, the authors subsequently explored the effect of SINO on the M1/M2 polarization of AMs in ALI. The numbers of M1 and M2 AMs in the lung were detected by FCM. F4/80 is the specific marker that distinguishes AMs from dendritic cells, iNOS and CD206 are the specific markers for M1 and M2 phenotypes of AMs, respectively. In the comparison of the control group, LPS significantly enhanced the expression of iNOS in AMs; meanwhile, LPS slightly decreased the expression of CD206 on the surface of AMs (Fig. 3A). After SINO pretreatment, the proportion of F4/80^+^iNOS^+^ AMs (M1 phenotype) decreased from 7.24 % to 2.34 %, while the proportion of F4/80^+^CD206^+^ AMs (M2 phenotype) increased from 12.8 % to 42.3 % (Fig. 3A).

Given the close relationship between the secretion of both IL-1β and IL-18 with pyroptosis, and the above experimental results that SINO could counteract the excessive production of IL-1β and IL-18 induced by LPS, the authors supposed that SINO has the potential to suppress the pyroptosis of AMs. Western blot showed that SINO was capable of significantly prohibiting the overexpression of pyroptosis-related biomarkers induced by LPS, which included NLPR3, cleaved-Caspase-1 (C—Caspase-1), ASC, IL-1β, and IL-18 (Fig. 3B). Together, such data indicated that pretreatment with SINO effectively mitigated the LPS-induced M1 polarization and pyroptosis of AMs in the lungs of mice.

### SINO partly reversed the inflammation, M1 polarization, and pyroptosis of AMs induced by LPS *in vitro*

**Fig. 4:**
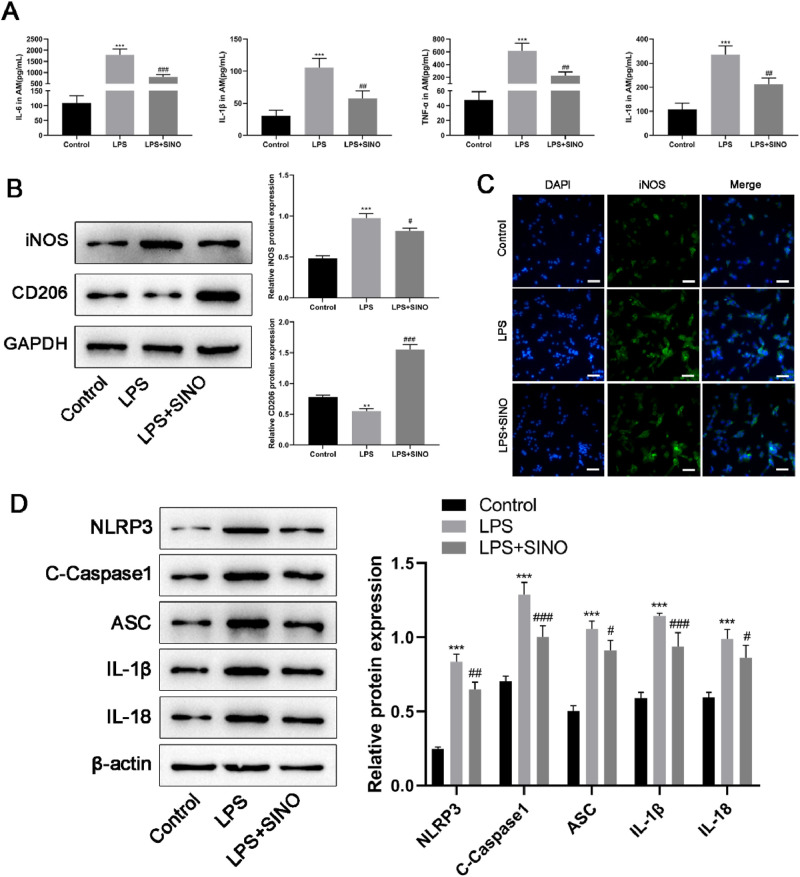
SINO partly reversed the inflammation, M1 polarization, and pyroptosis of AMs induced by LPS *in vitro*. The AMs were pretreated with SINO (10 μg/mL) for 1 h and then exposed to LPS (100 ng/mL) for 24 h. (A) The expression level of IL-6, IL-1β, TNF-α, and IL-18 in the supernatants of cultured AMs was determined using ELISA method. (B) The expression of iNOS and CD206 in AMs was detected by western blot. (C) Immunofluorescence was used to label the M1 phenotype (iNOS) of AMs. (D) The expression levels of pyroptosis-related proteins in AMs were detected by western blot. *** *p* < 0.005 vs. control group, ^###^*p* < 0.005 and ^##^*p* < 0.01 vs. LPS group.

The effects of SINO on the pro-inflammatory cytokine secretion, M1/M2 polarization, as well as pyroptosis of AMs *in vitro*, were also analyzed. Similar to the effects of SINO on the release of pro-inflammatory cytokines in ALI mice, *in vitro* analysis showed that SINO somehow reversed the increment of IL-6, IL-1β, TNF-α, and IL-18 induced by LPS (Fig. 4A). Western blot and immunofluorescence staining were exploited to investigate the M1 polarization of AMs, which showed that SINO could partly reduce the M1 polarization induced by LPS in AMs (Fig. 4B and C). More importantly, the authors found that the expression of CD206 in the LPS+SINO group was much stronger than that in the LPS group and even the control group (Fig. 4B), suggesting that SINO has a potent effect on stimulating M2 polarization *in vitro*. Moreover, the expression levels of NLPR3, C—Caspase-1, ASC, IL-1β, and IL-18 in AMs were significantly elevated following LPS stimulation (Fig. 4D). On the other hand, the SINO pretreatment could mitigate this increment induced by LPS (Fig. 4D). Collectively, these results suggested that SINO could also regulate the inflammation, M1/M2 polarization, and pyroptosis of AMs *in vitro*.

### SINO suppressed LPS-induced inflammation, M1 polarization, and pyroptosis of AMs by blocking NF-κB signaling

**Fig. 5:**
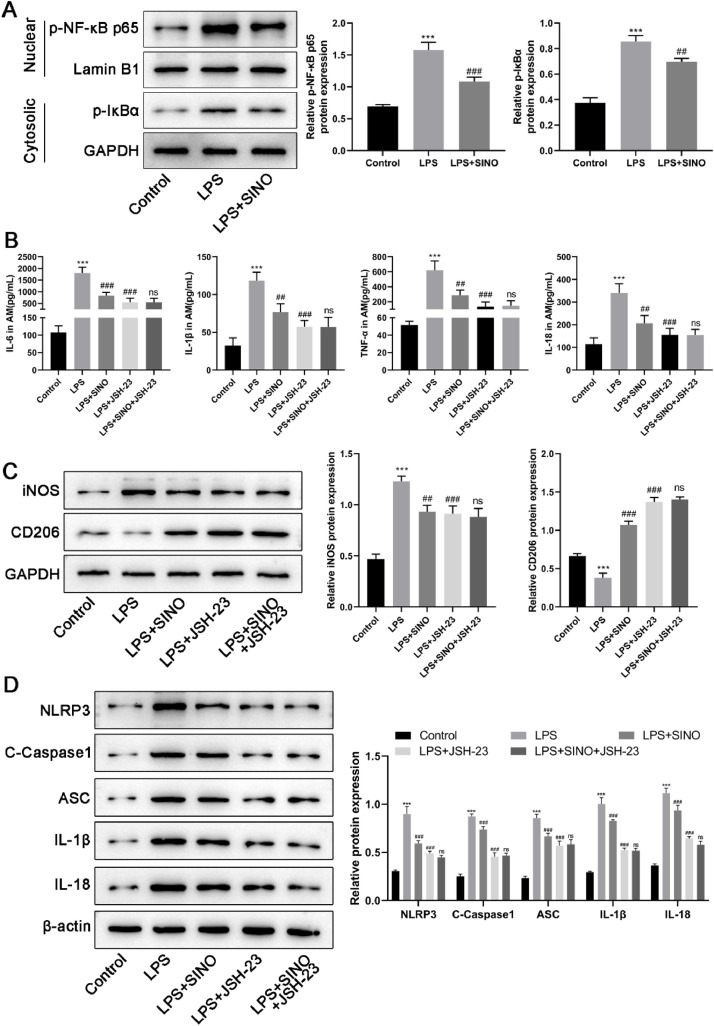
The effect of SINO on the inflammation, polarization, and pyroptosis of AMs was mediated by NF-κB signaling. The AMs were pretreated with SINO (10 μg/mL) for 1 h and then exposed to LPS (100 ng/mL) for 24 h. (A) The expression and phosphorylation levels of NF-κB p65 and IκBα was detected by western blot. The AMs were pretreated with SINO (10 μg/mL) or JSH-23 (inhibitor of NF-κB signaling) alone or their combination for 1 h, followed by LPS (100 ng/mL) stimulation for 24 h. (B) IL-6, IL-1β, TNF-α, and IL-18 levels in the supernatants of cultured AMs was determined using the ELISA method. (C) The expression of iNOS and CD206 in AMs was detected by western blot. (D) The expression levels of pyroptosis-related proteins in AMs were detected by Western blot. *** *p* < 0.005 vs. control group, ^###^*p* < 0.005 and ^##^*p* < 0.01 vs. LPS group, ns, not significant *vs.* SINO group.

Increasing studies have implicated the NF-κB signaling in the progression of ALI since NF-κB is a critical regulator of inflammatory response, pro-inflammatory genes that are involved in the inflammatory response^20^. It has been reported that SINO functions as a blocker of the NF-κB activation. So, the authors further explored whether the suppressive effect of SINO on LPS-induced inflammation, M1 polarization, and pyroptosis of AMs involves the NF-κB signaling pathway. LPS stimulation led to a significant increase in the phosphorylation levels of cytosolic IκBα and nuclear NF-κB p65 in AMs, whereas pretreatment with SINO reduced the LPS-induced increasing levels of cytoplasmic IκBα and nuclear NF-κB p65 phosphorylation (Fig. 5A). This data revealed that SINO could down-regulate the NF-κB signaling in LPS-induced AMs.

To further validate the involvement of NF-κB signaling in the protective effects of SINO, JSH-23, a selective inhibitor of NF-κB nuclear translocation, was employed as a pharmacological control. Treatment with LPS + JSH-23 significantly attenuated the secretion of proinflammatory cytokines, including IL-6, IL-1β, TNF-α, and IL-18, in AMs (Fig. 5B). In addition, JSH-23 suppressed the expression of the M1 marker iNOS, while partially restoring the M2 marker CD206 (Fig. 5C). Moreover, pyroptosis-associated proteins, such as NLRP3, cleaved caspase-1, ASC, IL-1β, and IL-18, were markedly reduced by JSH-23 treatment (Fig. 5D). Notably, treatment of SINO combining with JSH-23 did not confer additional inhibitory effects compared to either agent alone, suggesting that SINO may exert its anti-inflammatory, polarization-modulating, and anti-pyroptotic effects via suppression of the NF-κB signaling pathway.

## Discussion

SINO has been reported to potently control multiple inflammatory diseases^21^^,^^22^, especially rheumatoid arthritis;^12^ however, its role and underlying mechanism in ALI are largely unclear. LPS is widely known as a pivotal component causing ALI, which could elicit an acute inflammatory response in the lungs that simulates many clinical features of ALI^23^. Therefore, the LPS-induced ALI mouse model is considered a representative model to explore novel therapeutic strategies for human ALI^24^^,^^25^. In this paper, the authors found that SINO exerts protective action in LPS-induced ALI mice, as revealed by survival analysis and pathological changes in the lungs. Upon stimulation with LPS, pulmonary neutrophils were accumulated, and the pivotal pro-inflammatory cytokines are initiated to enhance inflammatory responses, thereby contributing to the progression of ALI^26^^,^^27^. The data showed that LPS stimulation led to systemic inflammation in mice, as evidenced by the enhanced production of IL-6, IL-1β, TNF-α, and IL-18, and the increase of neutrophils. SINO pretreatment effectively blocked these increases, which was in line with the finding of a previous study that SINO significantly reduced the expression levels of the pro-inflammatory cytokines and neutrophil infiltration in LPS-induced ALI mice^16^. An increase of MPO activity in the lungs is another feature of ALI, which could indirectly reflect the degree of oxidant stress, neutrophil infiltration, as well as pulmonary edema with high protein content during ALI^28^. Unsurprisingly, SINO could also attenuate LPS-induced ALI by decreasing the MPO activity and total protein content in the lung. These data confirmed the therapeutic potential of SINO in ALI.

A growing body of evidence supports that AMs contribute to the pathogenesis of ALI^5^. After LPS invades the pulmonary microenvironment, a portion of AMs are rapidly polarized toward the M1 phenotype to function as pro-inflammatory regulators during the development of ALI^29^^,^^30^. Hence, the M1 phenotype of AMs is considered to be toxic, while the M2 phenotype of AMs is characterized as protective during ALI^7^. Several reports indicated that the anti-inflammatory activity of SINO may be partly attributed to its regulatory role in macrophage polarization. Shi et al. found that SINO attenuates inflammatory injury in intracerebral hemorrhage by stimulating microglia M2 polarization^31^. A recent study revealed that SINO could promote macrophage reprogramming toward M2 phenotype to protect mice from endotoxemia^32^. However, whether SINO has a similar effect on AMs in ALI is unraveled. Interestingly, the current study revealed that SINO could regulate polarization of the AMs in LPS-induced ALI mice, as characterized by repressing M1 polarization and stimulating M2 polarization.

In the last decades, pyroptosis, an inflammatory form of cell death, has also been proven to be responsible for the progression of ALI. LPS stimulation causes the formation of the NLRP3/ASC/Caspase-1 inflammasome complex, thereby activating the pyroptosis of AMs in the lung^17^. Similarly, the authors also found that pyroptosis occurred in the lung tissues of mice after exposing LPS for 24 h, as evidenced by a significant up-regulation of inflammatory cytokines and activated NLRP3/Caspase-1 pathway-associated genes. In a recent study, SINO suppressed the pyroptosis of cardiomyocytes by blocking the activation of NLRP3 inflammasome with the reduction of NLRP3 and ASC expressions^33^. The present data suggested that SINO could effectively restrain the LPS-induced increase of NLPR3, C—Caspase-1, ASC, IL-1β, and IL-18 expression *in vivo*. These findings provided evidence that SINO protected against LPS-induced ALI, probably by its role in inhibiting the activation of M1 and NLRP3 inflammasome in AMs.

To explore the mechanism behind the protection of SINO in ALI, further *in vitro* experiments were designed and carried out. The *in vitro* study demonstrated that SINO also affects pro-inflammatory cytokine secretion, polarization, and pyroptosis at the cellular level. NF-κB, a heterodimeric protein composed of p65 and p50 subunits, is widely known to regulate the production and release of inflammatory cytokines^34^. During lung inflammation, the activation of NF-κB signaling has been observed in multiple pulmonary cells, including epithelial cells^35^, neutrophils^36^, as well as AMs^37^. The present study provided *in vitro* evidence showing that SINO prevented NF-κB activation in LPS-induced AMs as revealed by down-regulation of the phosphorylation of NF-κB p65 and its inhibitory factor IκBα, which suggested that NF-κB inactivation was involved in the mechanism of SINO on LPS-induced ALI. To validate the involvement of NF-κB signaling in SINO-mediated protection, the authors introduced JSH-23, a specific NF-κB inhibitor. Notably, JSH-23 alone effectively suppressed LPS-induced inflammation, M1 polarization, and pyroptosis in AMs, with similar efficacy to SINO. Moreover, co-treatment with SINO and JSH-23 did not produce additional inhibitory effects, suggesting that SINO exerts its protective effects at least in part by targeting the NF-κB pathway. These *in vivo* and *in vitro* results provide strong evidence that the inhibition of NF-κB activation is a central mechanism by which SINO mitigates ALI pathogenesis.

Based on these data, SINO could not only protect against LPS-induced ALI but also attenuate the associated inflammatory responses, M1 macrophage polarization, and pyroptosis of AMs by suppressing NF-κB signaling. Nevertheless, there are some limitations in this study. First, the present findings in the *in vivo* experiments do not exclude the possibility that SINO may have effects in other cells, such as epithelial cells. Secondly, the molecular mechanisms by which SINO inhibits NF-κB signaling in LPS-induced ALI require further work.

## Conclusion

Collectively, the present study demonstrated that SINO could significantly reduce M1 polarization and pyroptosis of AMs by NF-κB signaling, thereby attenuating inflammatory responses in the lung and further mitigating LPS-stimulated ALI. The present study lays a favorable mechanistic foundation for exploring the therapeutic potential of SINO in ALI.

## Authors’ contributions

Xiaoli Jian, Liang Zhao, Tingyun Yuan and Feng Li conceived and designed the project, Jiawu Yang, Peilong Li and Chuxiong Gong acquired the data, Qiong Li, Zhu Tian and Enli Lan analysed and interpreted the data, Xiaoli Jian and Liang Zhao wrote the paper, Tingyun Yuan and Feng Li revised the manuscript. All authors read and approved the final manuscript.

## Funding

This work was supported by Kunming Spring City Plan Specialized Research Fund for Young Talents.

## Data availability

The data that support the findings of this study are available from the corresponding author upon reasonable request.

## Declaration of competing interest

The authors declare no conflicts of interest.

## Appendix Supplementary materials

Image, application 1
