# Supplementary material for: Sinomenine ameliorates lipopolysaccharide-induced acute lung injury by stimulating M2 polarization and suppressing pyroptosis in alveolar macrophages
Source: Clinics (Sao Paulo). 2025 Nov 19;80:100832. doi: 10.1016/j.clinsp.2025.100832 (PMC12670450; doi:10.1016/j.clinsp.2025.100832)
Supplement: Supplementary file 1 [file mmc1.docx]

CLINICS-D-25-00428_Supplemnetary Material

**Table S1** The information of antibodies used in Western blot.

| **Antibody** | **Manufacturers** | **Cat.no** |
| --- | --- | --- |
| NLRP3 | Abcam | ab263899 |
| Cleaved-Caspase 1 |  | ab138483 |
| ASC |  | ab70627 |
| IL-1β |  | ab254360 |
| IL-18 |  | ab239611 |
| β-actin |  | ab8226 |
| iNOS |  | ab210823 |
| CD206 |  | ab64693 |
| GAPDH |  | ab8245 |
| NF-κB p65 |  | ab207297 |
| p-NF-κB p65 |  | ab239882 |
| Lamin B1 |  | ab16048 |
| IκBα |  | ab109300 |
| p-IκBα |  | ab283319 |
